# An approach to addressing governance from a health system framework perspective

**DOI:** 10.1186/1472-698X-11-13

**Published:** 2011-12-02

**Authors:** Inez Mikkelsen-Lopez, Kaspar Wyss, Don de Savigny

**Affiliations:** 1 Swiss Tropical and Public Health Institute, Basel, Switzerland; 2 University of Basel, Basel, Switzerland

## Abstract

As countries strive to strengthen their health systems in resource constrained contexts, policy makers need to know how best to improve the performance of their health systems. To aid these decisions, health system stewards should have a good understanding of how health systems operate in order to govern them appropriately. While a number of frameworks for assessing governance in the health sector have been proposed, their application is often hindered by unrealistic indicators or they are overly complex resulting in limited empirical work on governance in health systems. This paper reviews contemporary health sector frameworks which have focused on defining and developing indicators to assess governance in the health sector. Based on these, we propose a simplified approach to look at governance within a common health system framework which encourages stewards to take a systematic perspective when assessing governance. Although systems thinking is not unique to health, examples of its application within health systems has been limited. We also provide an example of how this approach could be applied to illuminate areas of governance weaknesses which are potentially addressable by targeted interventions and policies. This approach is built largely on prior literature, but is original in that it is problem-driven and promotes an outward application taking into consideration the major health system building blocks at various levels in order to ensure a more complete assessment of a governance issue rather than a simple input-output approach. Based on an assessment of contemporary literature we propose a practical approach which we believe will facilitate a more comprehensive assessment of governance in health systems leading to the development of governance interventions to strengthen system performance and improve health as a basic human right.

## Governance in the health sector

Low- and middle-income countries are in an era of unprecedented expansion of financial resources for health, both from development assistance and government spending [1]. However, during the recent financial crisis, many donors and governments cut back funding for health [2], requiring health system stewards to pay more attention to the traceability of fund allocations. Although funding levels can significantly influence health system performance, a large part of variation in health system performance across countries cannot be entirely explained by conventional factors such resource allocation (financial, human, technical). Rather, a deeper exploration of governance mechanisms such as the formal rules and informal customs could explain some of these differences. Governance has been studied in various dimensions from global governance [3,4], governance of the private sector in offering public services [5], corporate governance [6] and governance for development [7,8] There has also been an increasing interest in understanding the relationship between governance and health at the global level though discussions on global health governance (GHG) [9-12], together with developing theoretical frameworks for defining and measuring general governance [8,13,14]. Corresponding to this, there has been an increased interest in the assessment of governance in the health sector which is particularly important considering the characteristics of the health sector such as asymmetry of information and influence among the growing number of health system stakeholders [15] who have specific interests and different positions of power which may affect policy development [16]. This is particularly dynamic over the past decade with the rapid growth in the number of global health initiatives and their agents at country level. Therefore much conceptual thinking has gone into governance, especially from a political science perspective; however, it is not the intention of this paper to further contribute to the discourse in this area as, at least for health, this has been done by others [17-19]. Instead we aim to provide examples of how these often theoretical considerations could be applied to health system governance. We build on previous literature to develop a modified approach to assess select governance elements within the health system with a view to guiding health system-level interventions. This approach is aimed towards health sector stewards and practitioners who wish to understand potential governance issues within their health system and require a practical tool to do so.

## Governance in health systems

Furthering the discourse on governance is important as this topic is often neglected in international and national debates due to its complex and sometimes sensitive nature. The complex nature is underlined by the numerous definitions of governance and how it differs from management. We use the WHO (2007) definition of governance which is "*ensuring strategic policy frameworks exist and are combined with effective oversight, coalition-building, the provision of appropriate regulations and incentives, attention to system-design, and accountability*"[20]. Therefore, good governance from that perspective is understood to be policy- centric including consideration of all actors who impact the health system together with the various incentives which influence or regulate the system and stakeholder behaviours, though transparent rules overseen by strong accountability links. Improving the understanding of governance is especially important in less developed countries whose health systems are sometimes congested by numerous externally driven health initiatives who do not necessarily work together or respect country priorities [21] and who need to manage a plethora of stakeholders who influence policies. Governance also incorporates management which is concerned with implementing policies and decisions [18]. The importance of governance in health systems is evident from the fact that most conceptualisations and descriptions of health systems developed over the past decade speak of aspects of governance, either in terms of stewardship, regulation, oversight or governance itself (Table 1 - Chronology of major health system definitions, frameworks and concepts).

**Table 1:** Chronology of major health system definitions, frameworks and concepts

Conceptualisation	Main governance aspects	Reference
Health System Performance	First emphasis on stewardship as a health system function	[52]
Essential Public Health Functions	Strengthening public health regulation and enforcement capacity as one of the eleven essential public health function	[53]
Control Knobs	Regulation as one of the health system control knobs to improve performance	[54]
Strengthening Health Systems	Strengthening health system capacity by focusing on stewardship and regulation	[55]
Health System Building blocks	Articulation of governance as one of the six major building blocks of the health system, and rephrasing stewardship into governance	[20]
Health Systems Dynamics	Identifying stewardship and organizational arrangements as one of the four levers available to policy makers to achieve objectives and goals	[56]
Maximizing positive synergies	Ensuring that governance along with the other six functions of a health system are driven by people to promote equity	[23]
Systems thinking for Health Systems Strengthening	Links system thinking to health system building blocks, and conceptualizes governance across the building blocks.	[25]
Monitoring Building Blocks of the Health System	Proposes indicators for monitoring governance and the other building blocks of the health system	[43]

One of the most well known and provocative contribution to the health system discourse is the 2000 World Health Organisation's World Health Report on 'Health Systems: Improving Performance'. In this report, the health system was defined as "*all activities whose primary purpose is to promote, restore or maintain health*" and was presented as having four functions: stewardship; resource generation; financing; and service provision. Governance is included under the concept of stewardship which in turn was defined as "*the careful and responsible management of the well-being of the population*". The objectives of the health system were defined as: 1) improving the health of the population they serve; 2) responding to people's expectations; and 3) providing financial protection against the cost of ill health [22]. The WHO 2000 health system framework was later updated in 2007 with the release of the WHO report *'Everybody's Business: Strengthening Health Systems to Improve Health Outcomes: WHO's Framework for Action*' where the health system architecture was further elaborated as having six building blocks: leadership and governance; health workforce; information; medical products, vaccines and technologies; financing; and service delivery [20]. Here, governance was proposed as *"ensuring that strategic policy frameworks exist and are combined with effective oversight, coalition building, regulation, attention to system-design and accountability"*. A year later, the WHO further developed their conceptual framework for primary health care by placing people in the centre of the health system [23]. People are vital to the functioning of a health system, both benefiting from it and contributing to it as tax payers and also co-producers of health by adopting certain lifestyle choices [24]. A further refinement of the WHO 2007 framework was proposed by de Savigny and Adam (2009) who highlighted the importance of incorporating a systems thinking view of the synergies and interactions among and across all building blocks in the health system [25]. They point out that governance operates in its own right in the system as well as in every other building block. This is important as any intervention in one building block of the health system is likely to have system-wide effects which may need to be mitigated or prevented. A systems thinking view point requires a deeper understanding of the complex interactions among the various stakeholders who may have different objectives and power levels, and how decisions may affect them. Beyond systems thinking in health, it is also important for stewards to recognise the role and impact of the health system in the broader socio-political environment and that health systems are themselves social determinates which can influence education and employment [26].

Thus, as the conceptualisation of health systems has evolved, so has a deepening of the understanding of the critical role of governance. However, approaches and methods to systematically assess governance in health systems remain scarce. In the following section, we review various studies which have focused on governance in health and highlight the substantial contributions which they have made towards our overall understanding of the importance of governance.

### How has governance in health systems been conceived so far?

A substantial number of studies have discussed the various effects of select aspects of governance on the health sector [17,27-36]. Furthermore, some studies have empirically assessed the magnitude and impact of certain governance elements on health sector performance [37-39]. In general, most of the literature on governance and health has focused on single elements of governance such as degree of government effectiveness, degree of corruption and community participation. They investigated these components against proxy indicators of health sector outcomes or performance such as immunization rates, percentage of low birth weight babies or child mortality. Although important in that they provide evidence of a relationship, these studies do not account for other potential governance elements which could affect the performance of a health system.

Defining governance within the health sector is still relatively new and the composition of governance varies across reports, suggesting that the conceptualisation of governance is an ongoing process. There are, however, a few common elements in governance as identified in Table 2 (Summary of governance elements as addressed in selected contemporary health literature).

**Table 2:** Summary of governance elements as addressed in selected contemporary health literature

*Governance element*	*Reference*			
	
	WHO 2007	Islam 2007	Siddiqi et al. 2009	Lewis & Pettersson 2009
**Accountability**	**●**	**●**	**●**	**●**

**Effectiveness/efficiency**			**●**	

**Equity**			**●**	

**Ethics**			**●**	

**Existence of standards**		○		**●**

**Incentives**	○			**●**

**Information/Intelligence**	**●**	**●**	**●**	**●**

**Participation/collaboration**	**●**	**●**	**●**	

**Policy/System Design**	**●**	**●**		

**Regulation**	**●**	**●**		

**Responsiveness**		**●**	**●**	

**Rule of Law**			**●**	

**Transparency**	○	○	**●**	○

**Vision/Direction**	○		**●**	

Key: ● indicates the governance element is identified as an discrete element

○ indicates the governance element is mentioned in context of other elements

The latest body of work on governance in health goes further into developing approaches to assess overall governance within the health system [40-43]. These examples suggest indicators which can be broadly divided into two groups: 1) determinants of governance; and 2) governance performance indicators [19]. Determinants of governance (or rule-based indicators as they are sometimes referred to [44]) describe whether a procedure, regulation, policy or law exists, whilst a governance performance indicator assesses to what degree rules or policies have been followed and enforced. In general, it is easier to obtain determinants indicators than performance indicators which usually require surveys such as Public Expenditure Tracking Surveys (PETS), facility surveys, exit interviews and household interviews.

Islam (2007) approaches the assessment of governance in the health system by using two summary components. The first is composed of the World Governance Indicators (WGI) [45] developed by the World Bank which rates a country on six governance dimensions: voice and accountability; political stability; governance effectiveness; rule of law; regulatory quality; and control of corruption, leading to an overall governance score for a country. The second component is health specific and breaks governance into five dimensions: information and assessment capacity; policy formulation and planning; social participation and system responsiveness; accountability; and regulation. It proposes a set of illustrative questions to be answered by key stakeholders such as how information is used, how government coordinates donor inputs and who participates in setting the policy agenda? This framework provides a comprehensive range of issues to explore and even provides suggestions on which stakeholders to interview. It has so far been applied in various countries including: Vietnam [46], Kenya [47] and Angola [48]. Common areas of 'weak' governance found were lack of participation, transparency and strategic vision.

Using a similar approach, WHO (2010) developed a toolkit to assess health systems which included a governance module where they divide the assessment of governance in the health system into either rules-based or outcome-based indicators. The rules-based indicators cover topics such as the existence of an essential medicines list and the existence of key health sector documents. The outcome-based indicators ask questions about the rate of stock-out or the proportion of informal payments. Both rule-based and outcome-based indicators are important. However a weakness of the WHO (2010) toolkit is that despite it being a 'health system toolkit', it asks questions that are limited to disease-specific or vertical programmes such as HIV/AIDS, reproductive health, malaria and tuberculosis, thus leaving out other key areas such as mental health. Furthermore, asking about the 'existence' of such policies says little about their implementation. At present, we can find no example where the WHO governance monitoring module has been applied.

Lewis and Pettersson (2009) developed a list of governance indicators for health systems grouped into five topics: budget management; human resources; institutional providers; informal payments; and institutions. Within each topic, groups of questions are proposed to investigate the topic in detail. For example, within human resources, questions include both governance determinants such as the existence of a licensing system for health care professionals, and performance based such as the frequency of illegal side-payments influencing hiring decisions, or the fraction of contracted staff not on site during visit. These indicators together with questions on the design of incentives allow the researcher to gain more in-depth understanding of the governance challenges for that particular topic. The indicators are generic enough to allow for comparisons and are a mix of those which can be obtained easily (such as the Country Policy and Institutional Assessment - CPIA index) and those which are more challenging such as the frequency of under-the-table payments. This framework too has not been applied in full in any country to date.

Another health-system specific governance framework was developed by Siddiqi et al (2009). The authors adapt the UNDP good governance concept [14] to produce a framework which encompasses ten health system governance principles to assess governance of the health system. For each principle, broad questions are proposed for both the national policy formulation level and at the implementation level. The analytical framework has been used for an assessment of health system governance in Pakistan and identified several areas of weakness such as lack of accountability at the national level and little strategic vision in designing policies.

Finally, there is also a sector-specific governance assessment toolkit ('Good Governance for Medicines') developed by WHO which focuses entirely on the pharmaceutical sector [33]. The principle goal of this assessment framework is to evaluate transparency in the sector and is accompanied by a guide on how to assess responses, thus reducing the possibility of subjective judgement. This assessment has been applied in 26 countries including: Bolivia; Cambodia; Jordan; Indonesia; Mongolia; and Papua New Guinea.

Most of these frameworks provide 'snapshots' of the state of governance in health systems by developing both quantitative and qualitative indicators. This is advantageous as they can highlight areas of possible gross weakness for example, if a country has no recent essential medicines list, or if there are irregularities in the payroll for health workers, or a lack of transparency in resource allocation. Some of these frameworks such as WHO (2010) and Lewis and Pettersson (2009) also permit cross-country comparisons which are useful at the international level. However, despite this information being useful for donors or international organisations, it is questionable whether it is useful for health system stewards who probably already know where such governance weaknesses are in their health systems and instead need to better understand why, where and how to intervene.

## Towards a new approach to assessing governance in health systems

**Figure 1:**
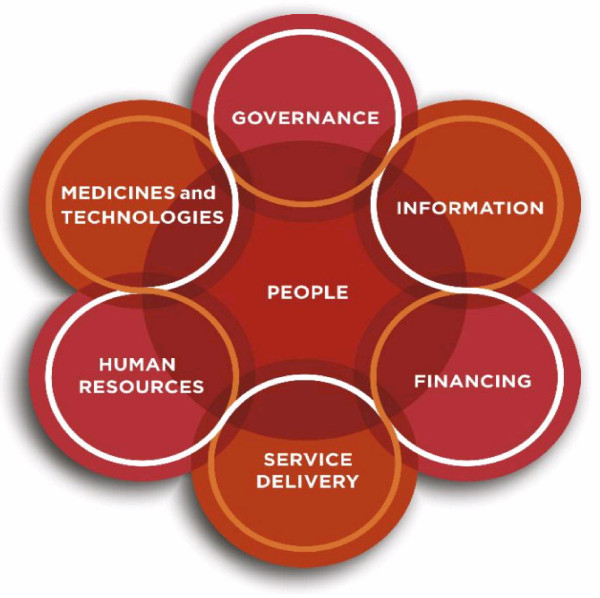
**Major interdependent health system building blocks. Reproduced with permission from de Savigny and Adam (2009)**.

For a governance framework to be of use to a health system steward it should: 1) be indicative of where governance issues are; 2) weight the individual elements composing governance in order to identify major drivers for "strong" or "weak" governance; and 3) provide a systematic way to assess these complexities. Our conceptual framework is based on the WHO (2007) model of the health system, but modified to adopt the systems thinking approach suggested by de Savigny and Adam (2009) where all the areas (or building blocks) intertwine (Figure 1 - Major interdependent health system building blocks).

For the purposes of extending a system-wide view of governance in the health system we appreciate that not all six building blocks are conceptually equivalent blocks. We see service delivery as a health system output and a primary interface for perceived quality of the health system. Conversely, the health workforce; information; medicines and technologies; and financing building blocks are health system inputs. As governance includes overseeing the entire health system, it permeates all other building blocks and is driven by people and actors in the system. This re-orientation of the WHO (2007) building blocks informs the basis of our framework.

**Figure 2:**
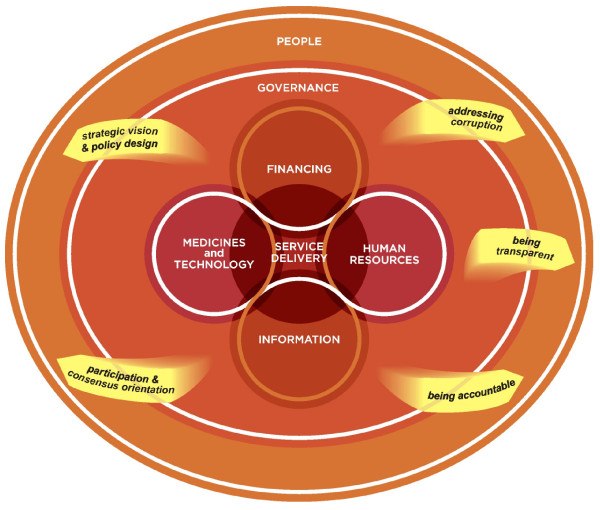
**Assessing governance across the health system**. Note: 'strategic vision & policy design' and 'participation & consensus orientation' can be viewed more conventionally as governance inputs, whilst 'addressing corruption', 'being transparent', and 'being accountable' are more governance processes.

In our approach (Figure 2 - An approach for assessing governance across the health system), we draw the most relevant and common governance elements found in Table 2 into a non-linear, systems thinking perspective on the health system. These elements can influence the functionality of the health system and can aid stewards to understand how the health system performs.

A vital element of good health system governance is the drive for long term *strategic vision *which is led by stewards using *transparent *information and which needs to be translated into appropriate policies with clear rules and correctly set incentives. A well designed system should increase integration and reduce fragmentation and duplication, and it should encourage *participation *of all relevant stakeholders, both state and non-state (such as citizen groups, pharmaceutical companies, insurance firms) in designing policies. As participation should include voices of numerous stakeholders which may not always be homogenous, health system stewards should strive to *seek consensus*. Although participation is encouraged, there are instances when too much participation could delay or even harm the delivery of health care [19]. It is also important for stewards to understand the possible informal influences which various stakeholders could be susceptible to and which could influence their voice. To ensure that the rules of the system are adhered to, a major process element of good health system governance is *being accountable*. Accountability involves "*holding public officials/service providers answerable for processes and outcomes and imposing sanctions if specified outcomes are not delivered*" [42]. More specifically, accountability requires identifying who has authority over what decisions and what their responsibilities include. It also includes how is transparent information on responsibilities, available resources and performance transmitted and used, and what incentives and sanctions are in place which may distort behaviours [19]. If all these elements are in place they can aid in addressing *corruption *"*misuse of entrusted power for private gain*"[29].

Although both 'regulation' and 'information' were common governance elements seen in Table 2, we did not include them as elements in their own right in our approach as creating information we believe is addressed in the information building block and since regulation includes addressing incentives, setting rules and enforcing them, we believe this is covered in accountability and system design.

In summary, a well governed health system should have clear goals based on a certain degree of participation of relevant stakeholders especially those from disadvantaged groups or who may have less power to influence polices, and from which transparent policies are designed and adhered to by promoting accountability and reducing the risk of corruption. Although we describe 'strategic vision & policy design' and 'participation & consensus orientation' as inputs and the others as processes, these are all interlinked within the governance building block and are dynamic and interchangeable. For example, improving accountability can be considered as an input to strengthening governance. Even an improvement in a single governance element would be an improvement in governance. For example, mitigating ways in which corruption can develop, or improving the transparency of budget allocation would both be considered an improvement in governance. However, these improvements in governance may not be sufficient to increase overall health system performance due to various non-governance factors which can influence overall health system performance [19]. Improved health system performance is a rather general term which could include various outcomes depending on the different interest groups within the health system. It could mean, for example, increased profit maximization for insurance companies, better effective coverage [49] for policy developers, increased responsiveness to the demands and needs of the population for citizen groups, or a general increased level and distribution of health.

### Example of an application

The starting point for the application of our approach would be to select an issue which impedes a health system outcome, for example limiting access and benefit from a public health care service. Various examples have been given above, so for purposes of illustration here we look at governance challenges in the health workforce, more specifically with regards to absenteeism. We recognise that there are other important governance challenges in the health workforce such as the migration of workers from rural to urban areas, or even at a global level which have been addressed by the 2010 WHO Global Code of Practice on International Recruitment of Health Personnel [50]. Absenteeism in a health system is an example of an issue which impedes timely access to health care services.

The ***first ***step the applier would need to do to understand why absenteeism could be occurring and persisting would be to map all the relevant stakeholders involved in human resource decisions and responses and what their roles, authorities, responsibilities and power relationships are [16]. This could be done by using the Policy Maker software which maps out the political dimensions of public policy and provides a guide for thinking about policy reform [51]. It is important to include as many stakeholders as possible as different stakeholders may see the reasons for absenteeism and the possible influence on the health system differently according to multi-finality [25].

The ***second ***step after stakeholder mapping is to work with stakeholders to identify areas where potential problems could be occurring. This can be done as a facilitated brainstorming looking for example at possible reasons for absenteeism through considering the design of the system, lines of authority to make decisions, the level inclusiveness of various groups in the design stage, or transparency of information and how it flows to those with managerial capacity. This can then be represented in a table according to our approach (the ***third ***step) Table 3 (Illustration of application of the approach: Considering the determinants for unauthorized health worker absenteeism in public facilities) illustrates this with an example (health worker absenteeism). As we encourage the assessment of governance throughout the health system, we have included a column for governance as the user also needs to assess the governance of the governing structures (such as health boards). This table guides the user across the approach to ensure that the various elements of governance are considered and how they manifest across the health system. It thus forces the user to take into account areas of the health system which they may not necessarily have considered. For example, the irregular flow of medicines and supplies could discourage health workers from being present.

**Table 3:** Illustration of application of framework: Considering the determinants for unauthorized health worker absenteeism in public facilities

Governance Element	Building Block				
	
	Financing Governance	Human Resources Governance	Information Governance	Medicines & Technology Governance	Governance
**Participation** **&** **Consensus Orientation**	Low participation from health workers in setting appropriate salary scales	Few ways of including community in health facility boards which provide oversight or advice to management.	Information on how community can participate in human resource decisions is not clear		Limited channels (such as health boards) for community or health workers to participate and have their voices heard

**Strategic Vision** **&** **System Design**	The system does not allow for incentives to be provided for working in less attractive areas; Salary increases not based on performance	No overtime payments structured in; Lack of performance appraisals; Distribution of staff is not based on service population making some environments more stressful	The design of the system does not require that data are regularly collected on staff attendance and transmitted to the district or above	Medicine delivery system is poorly designed leading to a lack of resources at health facility which make working environments less attractive as there are fewer resources available to staff.	System not designed to include sanctions that can be placed on health worker by management unit thereby reducing the ability to hold absent staff accountable

**Addressing corruption**	"Ghost" workers continually receive payments and are not identified by the system	Inability to replace ghost workers	Information on absenteeism is altered at health facility before it is transmitted, therefore hiding the problem	Absent staff may be taking publicly funded resources with them to sell in the private sector thus increasing incentive to be absent	Lack of supervision to ensure that health workers are present

**Being transparent**	Transparent information on salary scales and overtime payments is not available to staff	A list of staff who are supposed to be on call or at work is not available to the public	Information on staff attendance is not transferred to the authorities	Knowledge on future stock and flow is not transparent which could reduce the motivation for health staff to be present	Decisions made by health facility boards or management unit on hiring, promotions and firing are not made available to community

**Being accountable**	Ministry of Finance is not held to account when salary or bonus payments are late	Staff are not held to account when absent	No one is accountable for ensuring that regular, transparent data on staff attendance is collected and turned into information; Lack of information on sanctions options available to management unit to hold staff accountable	No one is held accountable if medicines go missing	Lack of enforcement options to hold absent staff accountable at the community or district level when staff are absent

The ***fourth ***step is a stakeholder assessment to examine this table and rank the most likely combination of tractable issues to be evaluated and identify the balance of incentives and disincentives which could explain the root cause of the problem (which may vary depending on the context). For example, the evaluators may find that the design of the system has not been adapted to recent health reforms (such as decentralisation) which will affect the balance of power and authority and may result in increased absenteeism in rural areas due to lack of supervision. This process could also aid in identifying the areas of strong governance which could be replicated in other areas of the system. The ***fifth ***step is to design an evaluation of the system-level governance interventions that follows the systems thinking approach [25] of combining process, contexts, effects and economic evaluation. For example, if absenteeism was consequent in part to a lack of supervision because following the decentralisation reform clear policies on supervisory responsibilities and sanctions on health staff absent without leave were not established, then the governance intervention would be to design clear policies on responsibilities and to ensure the authority and resources to implement them. In this example, the direct outcome for the health system of having unnecessary staff absenteeism at public health facilities will be reduced services for patients which may result in longer waiting times and increased dissatisfaction with the health system. If there are limited alternatives in the public sector, patients may lose faith in the public system and turn to the private sector which is usually more expensive and generally even less regulated. This will have equity implications as the poorest segments of the community who may have benefited from free health care now need to purchase their care, or go without care at all. Once these reasons for absenteeism of health staff are identified and understood, the health system can respond by developing and implementing interventions that try to promote incentives which make being absent less attractive. The outcome of this will be to reduce the problem of absenteeism which should have positive consequences for the health system.

### Differences between the approaches to assess governance in health systems

Assessing and understanding governance in the health system is crucial as public officials, donors and researchers strive to understand how to improve the performance of health systems. The concept of governance in health systems has evolved from a complex and often neglected issue in health policy debates to one which now features regularly in discussions and has motivated new research. Our approach draws heavily on prior work but differs in that it takes a problem-driven, system-wide approach and suggests a practical way to look at governance concerns through the WHO (2007) building block framework. It is designed to start from a certain governance issue which constrains the health system in performing to its optimum capacity, for example informal payments or unaccounted losses of essential medicines. In this way, our approach follows that of Savedoff (2009) who suggests that for assessment of governance in the health sector, a particular unit of analysis must be identified to focus the attention on relationships and issues which matter [19]. However, it differs in that our starting point is not necessarily an organisation or unit, but a problem which may involve various dimensions across building blocks of the system and therefore requires a broader assessment approach. Our approach guides the evaluator to assess comprehensively the various elements of the governance failure across the system. Like Siddiqi et al (2009) we also recommend that governance is assessed at different levels of the system such as the community, health facility, district, through to the national policy level and beyond [41], even considering the influence of other organisations such as unions, insurance companies and international partners who may profoundly affect the relationships and rules of the system. Depending on the initial starting point problem, the relevant importance of the different building blocks or governance elements may vary. For example, the relationships which are studied to understand the reasons for variations in medicine prices throughout a country will be different to those which look at whether recruitment is based on skills. If our approach is applied to various issues, it may illuminate common governance issues across various levels of the health system or common entry points for intervention. By promoting an outward driven assessment which includes all building blocks of the health system, our approach avoids reductionist thinking of only looking at input-output-outcome considerations for any given problem and encourages the applier to see the health system as a set of continuous and synergistic relationships. We recognise however, that there is no panacea to solve governance issues. This is an approach to improve and mitigate governance weaknesses but we do not propose that it would eliminate all governance bottlenecks.

A limitation of our approach is that as it doesn't provide a standard list of indicators. It does not allow comparisons between different contexts due to its broad nature, but it does take better account of the complexity of governance and is more flexible in that it includes all relevant aspects compared to other approaches based on standardised indicators. Our approach is also limited in that it must begin with an identifiable weakness in governance. This could be identified by applying one of the previous frameworks such as Islam 2007, Siddiqi et al (2009), or Lewis and Petterson (2009). Another limitation is that it only highlights where the barriers are and not how to design appropriate interventions. However by providing this first piece of the puzzle, stewards would be more informed and thus empowered to design interventions.

### Concluding remarks

In summary, based on an assessment of contemporary literature we propose this approach as a practical tool to facilitate the comprehensive assessment of governance in health systems which can be implemented by health system practitioners who are not necessarily specialists in governance analysis. This approach will help to identify the most promising entry points for system-level governance interventions and also contribute towards the appropriate design of policies taking into consideration the potential impact they have on the entire health system. This approach should also assist in advancing our understanding of governance in developing and transitional countries where health systems are often underperforming due to lack of investment, poor design and weak management practices, all of which can reduce the level of health care provided which is after all, a basic human right.

## Competing interests

The authors declare that they have no competing interests.

## Authors' contributions

IML, KW and DDS contributed equally to the conceptualisation and design of the approach. IML wrote the manuscript and all authors reviewed, contributed to, and approved the final manuscript.

## Pre-publication history

The pre-publication history for this paper can be accessed here:

http://www.biomedcentral.com/1472-698X/11/13/prepub

## References

[B1] Institute for Health Metrics and Evaluation Financing Global Health 2010: Development assistance and country spending in economic uncertainty 2010 Seattle WA, IHME

[B2] Feachem R Yamey G Schrade C A moment of truth for global health BMJ 2010 340 c2869 10.1136/bmj.c2869 20522664

[B3] Bradford CI Linn JF Global governance reform: Breaking the stalemate 2007 Washington DC: Brookings Institution Press

[B4] Finkelstein LS What Is Global Governance Global Governance 1995 1 367 372

[B5] Salamon LM Salamon LM The New Governance and the Tools of Public Action: An introduction The Tools of Government: A Guide to the New Governance 2002 New York: Oxford University Press 1 48

[B6] OECD OECD Principles of Corporate Governance 2004 Paris, OECD

[B7] de Ferranti D Ody AJ Jacinto J Ramshaw G How to improve governance 2009 Washington DC: Brookings Institution Press

[B8] Kaufmann D Kraay A Zoido-Lobaton P Governance Matters Policy Research Working Paper 1999 2196

[B9] Fidler DP Architecture amidst Anarchy: Global Health's Quest for Governance Global Health Governance 2007 1 1 17

[B10] Lee K Global health promotion: how can we strengthen governance and build effective strategies? Health Promotion International 2011 21 42 50 10.1093/heapro/dal050 17307956

[B11] Ng NY Ruger JP Global Health Governance at a Crossroads Global Health Governance 2011 3 1 37 PMC3983705 24729828

[B12] Hein W Burris SC Shearing CD Conceptual Models for Global Health Governance SSRN eLibrary

[B13] Arndt C Oman C Uses and Abuses of Governance Indicators 2006 Paris, Organisation for Economic Co-operation and Development

[B14] United Nations Development Programme Governance for Sustainable Human Development: a UNDP Policy Document 1997 New York, UN

[B15] Savedoff WD Transparency International The causes of corruption in the health sector: a focus on health care systems Global Corruption Report 2006 2006 London: Pluto Press

[B16] Walt G Gilson L Reforming the health sector in developing countries: the central role of policy analysis Health Policy Plan 1994 9 353 370 10.1093/heapol/9.4.353 10139469

[B17] Brinkerhoff DW Bossert TJ Health Governance: Concepts, Experience, and Programming Options 2008 Bethesda, MD, Health Systems 20/20

[B18] Fattore G Tediosi F Missoni E Attaining universal health coverage: the role of governance and management Attaining Universal Health Coverage, A research initiative to support evidence based advocay and policy making 2010 Milan: Egea 31 48

[B19] Savedoff WD Governance in the Health Sector: A Strategy for Measuring Determinants and Performance 2009 Portland, Maine, Social Insight

[B20] WHO Everybody's Business: Strengthening Health Systems to Improve Health Outcomes: WHO's Framework for Action 2007 Geneva, WHO 1 56

[B21] Ooms G Decoster K Miti K Rens S Van LL Vermeiren P Crowding out: are relations between international health aid and government health funding too complex to be captured in averages only? Lancet 2010 375 1403 1405 10.1016/S0140-6736(10)60207-3 20381858

[B22] WHO Health Systems: Improving Performance 2000 Geneva, World Health Organization. World Health Report

[B23] WHO Maximizing positive synergies between health systems WHO 2008 Geneva, WHO

[B24] Frenk J The Global Health System: Strengthening National Health Systems as the Next Step for Global Progress PLoS Med 2010 7 e1000089 10.1371/journal.pmed.1000089 20069038 PMC2797599

[B25] de Savigny D Adam T de Savigny D, Adam T Systems Thinking for Health Systems Strengthening 2009 Geneva, Alliance for Health Policy and Systems Research; WHO

[B26] WHO Commission On The Social Determinants Of Health Challenging inequity through health systems. Final Report - Knowledge Network on Health Systems 2007 Geneva, WHO Commission on the social determinants of Health

[B27] Brinkerhoff D Accountability and health systems: toward conceptual clarity and policy relevance Health Policy Plan 2004 19 371 379 10.1093/heapol/czh052 15459162

[B28] Chaudhury N Hammer J Kremer M Muralidharan K Halsey Rogers F Missing in Action: Teacher and Health Worker Absence in Developing Countries Journal of Economic Perspectives 2006 20 91 116 10.1257/089533006776526058 17162836

[B29] Transparency International: Global Corruption Report 2006: Health and Corruption 2006 Berlin

[B30] Vian T Review of corruption in the health sector: theory, methods and interventions Health Policy and Planning 2008 2 83 94 10.1093/heapol/czm048 18281310

[B31] The Global Fund to Fight AIDS Tuberculosis and Malaria Country coordinating mechanisms: Governance and civil society participation 2008 Geneva Switzerland, The Global Fund to Fight AIDS, Tuberculosis and Malaria. The Global Fund Implementer Series

[B32] Lagomarsino G Nachuk S Singh Kundra S Public stewardship of private providers in mixed health systems: Synthesis report from the Rockefeller Foundation - sponsored initiative on the role of the private sector in health systems 2009 Washington, DC, Results for Development Institute

[B33] WHO Measuring transparency in the public pharmaceutical sector: Assessment Instrument 2009 Geneva, WHO

[B34] Nishtar S Choked Pipes: Reforming Pakistan's Mixed Health System 2010 Oxford: Oxford University Press 20419962

[B35] Das Gupta M Khaleghian P Sarwal R Governance of Communicable Disease Control Services: A Case Study and Lessons from India SSRN eLibrary 2003

[B36] Ramiro LS Castillo FA Tan-Torres T Torres CE Tayag JG Talampas RG Community participation in local health boards in a decentralized setting: cases from the Philippines Health Policy Plan 2001 16 Suppl 2 61 69 11772991 10.1093/heapol/16.suppl_2.61

[B37] Rajkumar AS Swaroop V Public spending and outcomes: Does governance matter? Journal of Development Economics 2008 86 96 111 10.1016/j.jdeveco.2007.08.003

[B38] Gupta S Davoodi HR Tiongson ER Corruption and the Provision of Health Care and Education Services SSRN eLibrary 2000

[B39] Lewis M Governance and Corruption in Public Health Care Systems SSRN eLibrary 2006

[B40] Islam Med Health Systems Assessment Approach: A How-To Manual. Submitted to the U.S. Agency for International Development in collaboration with Health Systems 20/20, Partners for Health Reformplus, Quality Assurance Project, and Rational Pharmaceutical Management Plus 2007 Arlington, VA, Management Sciences for Health

[B41] Siddiqi S Masud TI Nishtar S Peters DH Sabri B Bile KM Framework for assessing governance of the health system in developing countries: gateway to good governance Health Policy 2009 90 13 25 10.1016/j.healthpol.2008.08.005 18838188

[B42] Lewis M Pettersson G Governance in Health Care Delivery: Raising Performance SSRN eLibrary 2009

[B43] WHO Monitoring the building blocks of health systems: a handbook of indicators and their measurement strategies 2010 Geneva, WHO

[B44] Kaufmann D Kraay A Governance Indicators: Where Are We, Where Should We Be Going? The World Bank Research Observer 2008 23 1 30

[B45] Kaufmann D Kraay A Mastruzzi M Governance Matters VIII: Aggregate and Individual Governance Indicators, 1996-2008 SSRN eLibrary 2009

[B46] Mai Oanh Tran Thi Van Tien Tran Huy Luong Duong Anh Tuan Khuong Khan Phuong Nguyen Cyong Le Quang Assessment of Health System Performance in Six Provinces Of Vietnam- Second Draft Report for Comments 2010 Bethesda MD, Health Systems 20/20 Project, Abt Associates Inc

[B47] Luoma M Doherty J Muchiri S Barasa T Hofler K Maniscalco L Kenya Health System Assessment 2010 2010 Bethesda, MD, Health Systems 20/20 project, Abt Associates Inc

[B48] Connor C Averbug D Miralles M Angola Health System Assessment 2010 2010 Bethesda, MD, Health Systems 20/20, Abt Associates Inc

[B49] Lozano R Soliz P Gakidou E bbott-Klafter J Feehan DM Vidal C [Benchmarking of performance of Mexican states with effective coverage] Salud Publica Mex 2007 49 Suppl 1 S53 S69 17469399 10.1590/s0036-36342007000700009

[B50] WHO WHO Global Code of Practice on the International Recruitment of Health Personnel Sixtry-Third World Health Assembly Agenda item 11.5 2010

[B51] http://polimap.books.officelive.com/default.aspx

[B52] WHO World Health Report 2000: Health Systems Improving Performance 2000 Geneva: WHO

[B53] Pan American Health Organization Essential public health functions Public health in the Americas. Scientific and technical publication no. 589 2002

[B54] Roberts M Hsiao W Berman P Reich M Getting Health Reform Right: A Guide to Improving Performance and Equity 2004 Oxford University Press

[B55] Mills A Rasheed F Tollman S Jamison DT, Breman JG, Measham AR, Alleyne G, Claeson M, Evans DB, et al Strengthening Health Systems Disease Control Priorities in Developing Countries 2006 2 New York: Oxford University Press 87 102

[B56] Atun R Menabde N Coker R, Atun R, McKee M Health systems and systems thinking Health Systems and the Challenge of Communicable Disease 2008 Open University Press

